# Effects of Integrated Extracts of *Trigonella foenum-graecum* and *Asparagus racemosus* on Hot Flash-like Symptoms in Ovariectomized Rats

**DOI:** 10.3390/antiox14030355

**Published:** 2025-03-18

**Authors:** Fusun Erten, Besir Er, Ramazan Ozmen, Muhammed Tokmak, Ebru Gokdere, Cemal Orhan, Abhijeet A. Morde, Muralidhara Padigaru, Kazim Sahin

**Affiliations:** 1 Department of Veterinary Science, Pertek Sakine Genc Vocational School, Munzur University, Tunceli 62500, Türkiye; fusunerten@munzur.edu.tr; 2 Department of Biology, Faculty of Science, Firat University, Elazig 23119, Türkiye; ber@firat.edu.tr; 3 Department of Animal Nutrition, Faculty of Veterinary Medicine, Firat University, Elazig 23119, Türkiye; rozmen@firat.edu.tr (R.O.); 211304205@firat.edu.tr (M.T.); corhan@firat.edu.tr (C.O.); 4 Department of Physiology, Faculty of Medicine, Firat University, Elazig 23119, Türkiye; egokdere@firat.edu.tr; 5 Research and Development, OmniActive Health Technologies Co., Ltd., Mumbai 400013, India; a.morde@omniactives.com (A.A.M.); m.padigaru@omniactives.com (M.P.)

**Keywords:** menopause, *Trigonella foenum-graecum*, *Asparagus racemosus*, menopausal symptoms, hormone therapy, vasomotor symptoms, ovariectomy

## Abstract

Vasomotor symptoms, such as hot flashes (HFs), commonly affect women during menopause, leading to a reduced quality of life. The current study evaluates the combined effect of active components *Asparagus racemosus* (AR) and *Trigonella foenum-graecum* (TFG) in a single oral formulation (IAT) for alleviating menopausal symptoms in ovariectomized rats. Following bilateral ovariectomy, the animals were randomly assigned to nine groups: (1) Control, (2) Ovariectomy (OVX), (3) OVX+TA1 (TA: Combination of Trigonella and Asparagus; TFG 30 mg/kg + AR 30 mg/kg), (4) OVX+TA2 (TFG 30 mg/kg + AR 15 mg/kg), (5) OVX+TA3 (TFG 15 mg/kg + AR 30 mg/kg), (6) OVX+TA4 (TFG 40 mg/kg + AR 30 mg/kg), (7) OVX+TA5 (TFG 30 mg/kg + AR 40 mg/kg), (8) OVX+IAT1 (IAT: Integrated Asparagus and Trigonella; TFG+AR integrated extract, 30 mg/kg), and (9) OVX+IAT2 (TFG+AR integrated extract, 60 mg/kg). On the 8th day of treatment, tail and skin temperatures were recorded every 30 min for 24 h. Ovariectomized rats exhibited menopausal symptoms, such as hormonal imbalances and elevated skin temperature. Administration of AR, TFG, and IAT significantly decreased serum follicle-stimulating hormone (FSH), luteinizing hormone (LH), and cortisol while increasing estradiol, progesterone, and dopamine (*p* < 0.0001), effectively alleviating hot flash-like symptoms. Additionally, they mitigated ovariectomy-induced oxidative stress by lowering malondialdehyde (MDA) levels and restoring antioxidant enzyme activity. Ovariectomized rats exhibited increased expression of a proto-oncogene (c-FOS), gonadotropin-releasing hormone (GnRH), Kisspeptin, Neurokinin B (NKB), and Transient receptor potential vanilloid 1 (TRPV1), along with reduced expressing brain-derived neurotrophic factor (BDNF) levels, which were reversed by treatment, especially with the IAT2 combination. The AR and TFG combination, particularly in IAT formulations, showed strong potential in alleviating menopausal symptoms in ovariectomized rats. These findings suggest that the combination of AR and TFG extracts could be a natural alternative for managing postmenopausal symptoms by restoring reproductive hormone levels, regulating lipid profiles, and enhancing antioxidant defense systems.

## 1. Introduction

Menopause is a natural aging process characterized by the permanent cessation of menstruation due to follicular function loss [1]. Each day, approximately 6000 American women enter menopause, with around 75% of women aged 50–55 experiencing postmenopausal conditions [2,3]. The decline in circulating estrogen levels during menopause contributes to vasomotor symptoms, palpitations, sleep disturbances, headaches, fatigue, mood changes, and anxiety. Long-term consequences may include cardiovascular disease, osteoporosis, and cognitive decline [4,5]. However, vasomotor symptoms associated with hot flashes (HFs) and mood swings leading to poor quality of life are considered the most bothersome symptoms experienced by most women who look for medical intervention [6].

The decrease in estrogen triggers vasomotor symptoms such as HFs and night sweats in most menopausal women [7,8]. HFs result from disrupted thermoregulation in the hypothalamus, leading to increased skin blood flow and sweating [9]. HFs are closely associated with luteinizing hormone (LH) pulses, controlled by the hypothalamic neural circuit and pulsatile gonadotropin-releasing hormone (GnRH) secretion [8]. The pulsatile GnRH secretion is, in turn, controlled by a subpopulation of neurons that express Kisspeptin/Neurokinin B/Dynorphin (KNDγ) proteins [10]. Kisspeptin/Neurokinin B/Dynorphin neurons (KNDγ neurons), originating from the infundibular nucleus of the hypothalamus, act through the kisspeptin neuropeptide in the negative feedback loop of estrogens and GnRH [11,12]. Estrogen affects KNDγ neurons both directly by binding to their receptors and indirectly by increasing β-endorphin production [13,14]. Neurokinin B and dynorphin exert an autoregulatory effect by affecting kisspeptin secretion [15]. Neurokinin B increases kisspeptin production via the neurokinin-3 receptor (NK3R). KNDγ neurons play a central role in thermoregulation and send signals to thermoregulatory nuclei in the preoptic area (POA), where neurokinin B is released [14,16,17]. Thus, KNDγ neurons provide a possible link between the decrease in estrogen levels and changes in thermoregulation during the menopausal transition [14]. Transient receptor potential vanilloid 1 (TRPV1) is a thermal sensory receptor, part of transient receptor potential channels, and has a wide distribution in the POA [18]. Capsaicin injection into the POA leads to a rapid cooling response that is not observed in TRPV1-gene knockout mice [19]. TRPV1 can cause severe hyperthermia when used as an analgesic in an inflammatory model [20]. These results suggest that TRPV1 may play a role in the process of thermoregulation in the POA [21]. Furthermore, the proto-oncogene (c-FOS) protein that controls the cellular signal of genes involved in growth, development, and various cellular activities is often used as an indicator of neural activity [22]. Additionally, estrogen plays a vital role in synthesizing and expressing brain-derived neurotrophic factor (BDNF), which regulates neuronal function in the hippocampus [23].

Hormone replacement therapy (HRT) remains the most effective treatment for menopausal vasomotor symptoms, such as HFs and night sweats [24]. However, long-term HRT use is associated with increased risks of hormone-related cancers and cardiovascular disease. For women who cannot or do not wish to use HRT, alternative treatments such as mood stabilizers and selective serotonin reuptake inhibitors (SSRIs) are sometimes recommended, though data on their safety and efficacy are limited [3,25]. Phytoestrogen-based supplements, including soy, red clover, flaxseed, hops, hesperidin, and kudzu, help to reduce the frequency, intensity, and number of daily vasomotor symptoms and improve the quality of life [26]. Additionally, phytoestrogens affect cellular signaling pathways to regulate collagen synthesis through intracellular estrogen receptors in skin fibroblasts [27].

*Asparagus racemosus* (AR), a plant from the Asparagaceae family, contains bioactive compounds such as steroidal saponins, alkaloids, quercetin, and glycosides of quercetin [28]. AR is an established adaptogen used in traditional medicine to treat conditions associated with the female reproductive system, including menopause, the immune system, and the nervous system. Steroidal saponins such as shatavarin IV from the root of AR have phytoestrogenic effects, which are responsible for improving menopausal symptoms [29,30]. *Trigonella foenum-graecum* (TFG), from the *Fabaceae* family, is widely cultivated in the Mediterranean, North Africa, and the Indian subcontinent for culinary and medicinal purposes [31]. It has been used to manage diabetes, dyslipidemia, and hormonal imbalances [31,32,33]. TFG extract is rich in steroidal saponins such as protodioscin, which has estrogenic properties beneficial for sexual health, lactation, bone density, and menopausal symptom relief [34]. Further supplementation with TFG seed extract is associated with increased serum estradiol levels and reduced symptoms of HFs in menopausal women [33,35]. This study aims to evaluate the protective effects of standardized AR and TFG extracts, as well as their integrated formulations, in alleviating menopausal symptoms using an ovariectomized (OVX) rat model. This study further explores the effect of these extracts on sex hormones and neuronal mediators involved in menopause regulation.

## 2. Materials and Methods

### 2.1. Animals

In this study, 63 female Sprague-Dawley rats (n = 7 in each group; age: 8 weeks old, 180–200 g) were used. All animals were individually housed under clean conditions with controlled temperature (22 ± 2 °C), humidity, and light (55 ± 5% relative humidity, 12 h light/12 h dark environment) and were provided with a standard commercial diet and water ad libitum (Table 1) [36]. All experiments were conducted in compliance with the National Institutes of Health Guide for the Care and Use of Laboratory Animals and EU directives.

After a week of adaptation, the rats underwent ovariectomy, while a control group underwent a procedure in which their skin and muscle tissue were opened and closed without ovariectomy. Prior to the ovariectomy procedure, the rats were administered intraperitoneal injections of 50 mg/kg ketamine hydrochloride and 8 mg/kg xylazine, which rendered them unconscious. The animals were first anesthetized, and their abdominal areas were shaved. To minimize the risk of infection, a sterile surgical drape was placed, and the surgical site was treated with a povidone-iodine solution. A laparotomy incision was then made to access the abdominal cavity. Once the ovaries were accessed, the mesovarium and tuba uterina were carefully ligated, and the bilateral ovarian tissues were surgically removed. After hemostasis had been achieved, the abdominal wall, subcutaneous tissues, and skin were sutured in layers, and the incision was closed. Following the OVX procedure, the incision sites were treated with the povidone-iodine solution for three days.

### 2.2. Study Design

Rats were randomly divided into nine groups 4 weeks after ovariectomy: (1) Control, (2) Ovariectomy (OVX), (3) OVX+TA1 (TFG, 30 and AR, 30 mg/kg BW), (4) OVX+TA2 (TFG, 30 and AR, 15 mg/kg BW), (5) OVX+TA3 (TFG, 15 and AR, 30 mg/kg BW), (6) OVX+TA4 (TFG, 40 and AR, 30 mg/kg BW), (7) OVX+TA5 (TFG, 30 and AR, 40 mg/kg BW), (8) OVX+IAT1 (integrated extract of TFG and AR at 30 mg/kg BW), and (9) OVX+IAT2 (integrated extract of TFG and AR at 60 mg/kg BW). The substances were administered orally via gavage at the specified doses. The dosage selection was based on previously published human clinical studies where Fenugreek and *Asparagus racemosus* were used at doses ranging from 500 to 1000 mg, showing significant benefits in postmenopausal symptoms [1,37]. The equivalent rat doses were calculated using allometric scaling to ensure biological relevance [38]. On the 8th day of the repeated administration, tail, core, and skin temperatures were measured every 30 min for a duration of 24 h. Blood and tissue samples were collected approximately two hours after the final administration. The extracts TFG, AR, and IAT were supplied by OmniActive Health Technologies (Mumbai, India).

The roots of AR were first extracted with 50% aqueous ethanol, followed by acetone extraction, yielding an acetone-soluble fraction containing 18–22% Shatavarin IV. TG seeds were extracted with 50% aqueous ethanol, and the ethanol-soluble fraction was concentrated to obtain 7.0–8.0% protodioscin. For the reparation of integrated extracts of AR and TFG, the hydro-alcoholic extract of a mixture of AR (roots) and TFG (seeds) in a 3:1 ratio was processed through styrene-divinylbenzene copolymer resin. The column was sequentially washed with water, followed by 30% ethanol, and elution with 90% ethanol. The ethanol fraction was then concentrated and dried into a powder containing 4% Protodioscin and 7% Shatavarin IV, as confirmed by HPLC analysis.

At the end of this study, all rats were decapitated via cervical dislocation, and blood and brain samples were collected. The serum was separated by centrifuging the blood at 3000× *g* for 10 min and stored at −80 °C until use.

### 2.3. Laboratory Analyses

#### 2.3.1. Biochemical Analyses

Serum glucose, triglyceride, cholesterol, aspartate aminotransferase (AST), alanine aminotransferase (ALT), and creatinine levels were measured with a portable automatic chemistry analyzer (Samsung LABGEO PT10, Samsung Electronics Co., Suwon, Republic of Korea). Serum follicle-stimulating hormone (FSH), luteinizing hormone (LH), 17β-Estradiol (E2), progesterone, cortisol and dopamine levels, and antioxidant enzyme parameters such as superoxide dismutase (SOD), catalase (CAT), and glutathione peroxidase (GSH-Px) were measured using relevant commercial enzyme-linked immunosorbent assay kits (ELISA, Elx-800, Bio-Tek Instruments Inc., Winooski, VT, USA). Commercially available kits (BT-LABS, Shanghai, China) were used to perform assays for SOD, CAT, and GSH-Px, following the manufacturer’s protocols. The detection limits for SOD (Cat. No. E2608Mo), CAT (Cat. No. E0076Mo), and GSH-Px (Cat. No. E2106Mo) were 0.1–35 ng/mL, 0.52–70 ng/mL, and 0.1–40 ng/mL, respectively. The intra-assay and inter-assay variability for all measured enzymes was kept below 8–10%.

Serum MDA levels were quantified by high-performance liquid chromatography (HPLC) according to the method described by Orhan et al. [39]. In brief, samples were homogenized with 0.5 M HClO_4_ and 2,6-di-tert-butyl-p-cresol, and the 20 µL of supernatant after centrifugation was loaded into the HPLC system (Kyoto, Japan), which consisted of an ultraviolet–visible (UV–vis) SPD-10 AVP detector, a CTO-10 AS VP column, a 30 mM KH_2_PO_4_: CH_3_OH (82.5:17.5, *v*/*v*, pH 3.6) mobile phase at a flow rate of 1 mL/min, and a 250 nm scanning wavelength.

#### 2.3.2. Western Blot Analysis

Western blot analysis was used to measure the protein levels of the c-FOS, GnRH, kisspeptin, NKB, BDNF, and TRPV1 in the brain tissues of rats. Brain tissue samples were homogenized in a 1:10 (*w*/*v*) solution containing 10 mM Tris-HCl (pH = 7.4), 0.1 mM PMSF, 0.1 mM NaCl, 5 μM soybean (Sigma, St. Louis, MO, USA), and 1% SDS. Homogenization was performed using a glass homogenizer, followed by sonication by respecting the cold chain using ice [40]. The homogenates were then centrifuged at 15,000 rpm for 60 min at a temperature of +4 °C. Protein denaturation was then carried out by adding an equal volume of sample buffer to the supernatant samples obtained after centrifugation. Protein quantification was performed using a NanoDrop microvolume spectrophotometer (Maestrogen Inc., Hsinchu, Taiwan). Following the measurement of protein concentrations, equal amounts of protein (20 μg) were subjected to sodium dodecyl sulfate-polyacrylamide gel electrophoresis and transferred to nitrocellulose membranes (Schleicher and Schuell Inc., Keene, NH, USA). The membranes were then blocked with 1% bovine serum albumin (BSA) and incubated with primary antibodies against TRPV1 (Cat. No. ab305299, Abcam, Cambridge, UK), kisspeptin (Cat. No. df7133, Affinity Biosciences, Cincinnati, OH, USA), NKB (Cat. No. pa5-110456, Thermo Fisher Scientific, Waltham, Massachusetts,, USA), c-FOS (Cat. No. ab190289, Abcam, Cambridge, UK), BDNF (Cat. No. ab108319, Abcam, Cambridge, UK), and GnRH (Cat. No. sc-25344) (Santa Cruz Biotechnology Inc., Dallas, TX, USA). The membranes were then washed five times for five minutes each with phosphate-buffered saline (PBS). Subsequently, nitrocellulose membranes were incubated with peroxidase-conjugated goat anti-mouse immunoglobulin (Cat. No. sc-2005, Santa Cruz Biotechnology Inc., Dallas, TX, USA). The visualization of the bands was achieved through the use of a diaminobenzidine solution. The relative intensities of the bands were analyzed using the ImageJ software program (version 1.52.a) developed by the National Institutes of Health (Bethesda, MD, USA).

### 2.4. Statistical Analysis

The SPSS statistical package program (IBM SPSS Version 22.0) was used for data analysis. The sample size (N = 63) was determined using the G*Power program (Version 3.1.9.3) with a power of 85%, an effect size of 0.56, and a significance level of 0.05 [41]. Based on these parameters, it was calculated that seven subjects per group would be sufficient. Prior to conducting parametric tests on the data, it was necessary to ascertain that the variances were homogeneous. This was achieved through the use of the Levene test. Additionally, the assumption of normality was evaluated through the Shapiro–Wilk test. A one-way analysis of variance (ANOVA) was used to determine differences between the groups. The variables are reported as mean ± standard error. A Tukey’s post hoc test was used for multiple comparisons. The cutoff value for a statistically significant difference was accepted as 0.05.

## 3. Results

### 3.1. Body and Tail Temperatures

**Figure 1:**
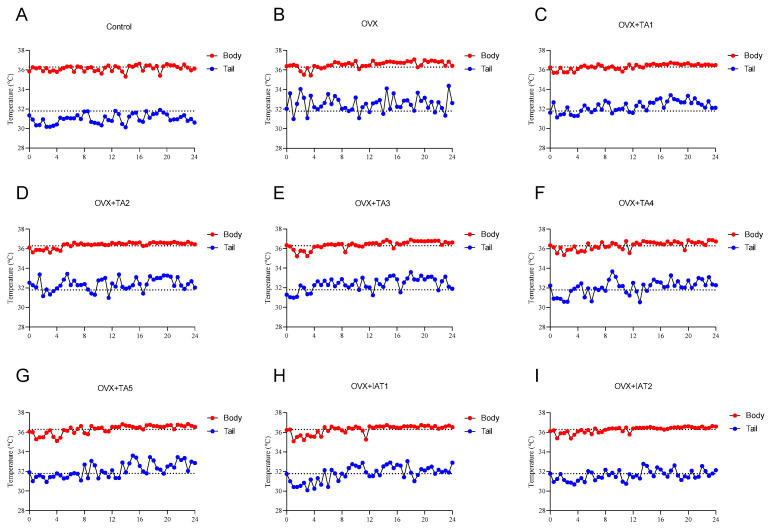
Effects of different combinations of *Trigonella foenum-graecum* (*TGF*) and *Asparagus racemosus* (*AR*) extracts on body and tail temperatures in OVX (Ovariectomy) rats (n = 7), where (**A**) represents the control group, (**B**) the OVX group, (**C**–**G**) different doses of TA combinations, and (**H**,**I**) integrated extract combinations. Group abbreviations: Control: on-ovariectomized rats without any treatment; OVX (Ovariectomy): Rats that underwent ovariectomy; OVX+TA1-TA5: rats treated with different dose combinations of *Trigonella foenum-graecum* (T) and *Asparagus racemosus* (A), OVX+IAT1-IAT2: rats treated with integrated combinations of T and A extracts integrated extract combinations.

**Figure 2:**
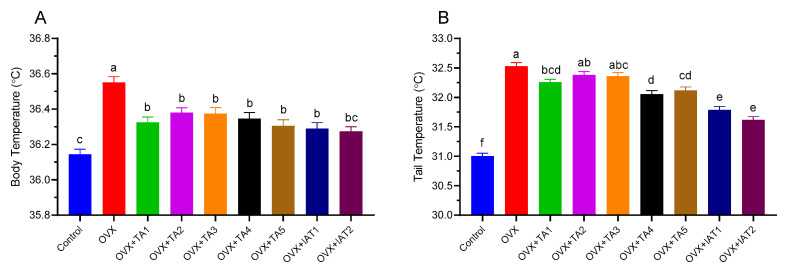
The effects of different combinations of *Trigonella foenum-graecum* (*TGF*) and *Asparagus racemosus* (*AR*) extracts on body and tail temperatures in OVX (Ovariectomy) rats (n = 7) in rats, where (**A**) represents the body temperatures, and (**B**) represents the tail temperatures. Group abbreviations: Control: on-ovariectomized rats without any treatment; OVX (Ovariectomy): Rats that underwent ovariectomy; OVX+TA1-TA5: rats treated with different dose combinations of *Trigonella foenum-graecum* (T) and *Asparagus racemosus* (A), OVX+IAT1-IAT2: rats treated with integrated combinations of T and A extracts integrated extract combinations. Effects of different combinations of *Trigonella foenum-graecum* (TFG) and *Asparagus racemosus* (AR) on body and tail temperatures in OVX rats (n = 7). Treatments lasted 8 days, with temperatures measured every 30 min on day 8. Error bars indicate the standard error of the mean. Data are presented as mean ± standard deviation. Different superscripts (a–f) denote significant differences (*p* < 0.05, ANOVA with Tukey’s post hoc test). Group abbreviations: Control, OVX (Ovariectomy), OVX+TA1-TA5 (different TFG+AR combinations), OVX+IAT1-IAT2 (integrated TFG+AR extracts).

Body and tail temperatures were recorded every 30 min for 24 h at room temperature (Figure 1 and Figure 2). The OVX group showed a significant increase in body temperature compared to the control group (Figure 2A; *p* < 0.0001). In contrast, groups treated with test products (TA1, TA2, TA3, TA4, TA5, IAT1, and IAT2) exhibited a slight but significant reduction in both body and tail temperatures compared to the OVX group (Figure 2A; *p* < 0.0001).

In the control group, body temperature ranged from 35.31 °C to 36.62 °C, with a mean of 36 °C and a maximum–minimum difference of 1.31 °C. The tail temperature ranged from 30.12 °C to 31.9 °C, with a mean of 31.1 °C and a range of 1.78 °C (Figure 1A). In the OVX group, body temperature ranged from 35.44 °C to 37.07 °C, with an average of 36.5 °C, showing no significant change compared to the control group (Figure 1B). However, the tail temperature increased, with a maximum of 34.37 °C, a minimum of 30.98 °C, and an average of 32.55 °C (Figure 1B). Additionally, OVX rats exhibited increased body temperature compared to the control group (Figure 1C–I), while treatment with plant extracts led to a reduction in tail temperature compared to OVX rats (Figure 1B–I).

### 3.2. Body Weight and Feed Intake

**Figure 3:**
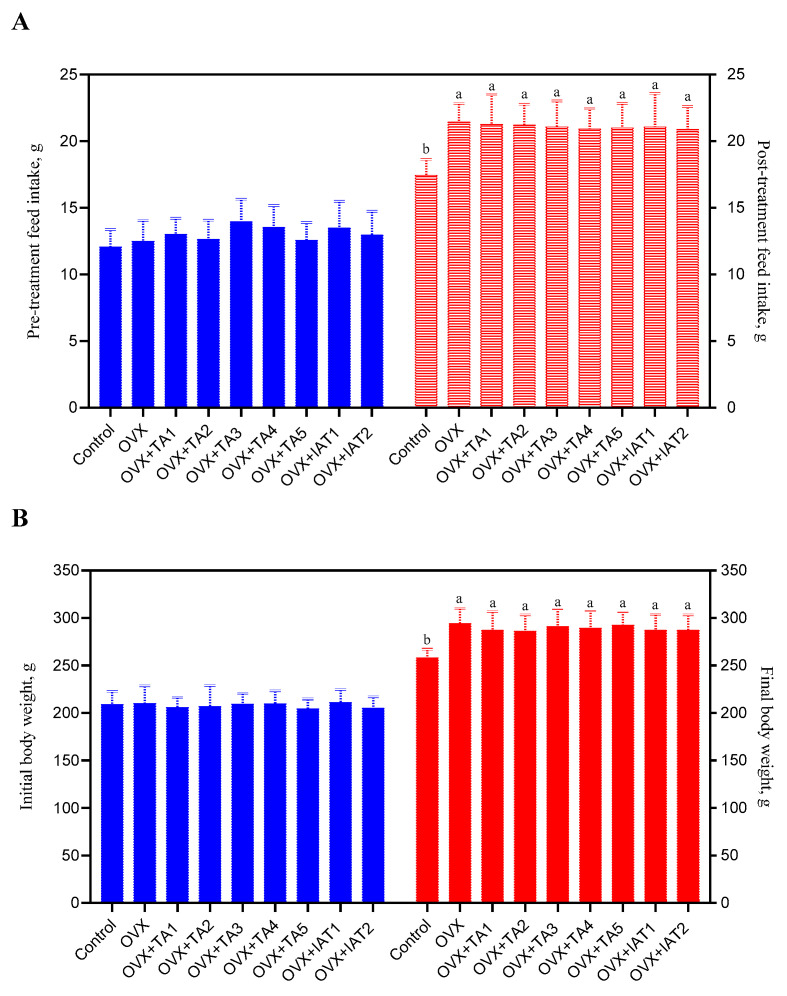
The effects of *Trigonella foenum-graecum* (TFG) and *Asparagus racemosus* (AR) combinations on feed intake (**A**) and body weight (**B**) in ovariectomized (OVX) rats (n = 7) are shown. Whisker plots represent the median and minimum–maximum values, with different letters (a, b) indicating statistically significant differences (ANOVA, Tukey’s test). Group abbreviations: Control: on-ovariectomized rats without any treatment; OVX (Ovariectomy): Rats that underwent ovariectomy; OVX+TA1-TA5: rats treated with different dose combinations of Trigonella foenum-graecum (T) and Asparagus racemosus (A), OVX+IAT1-IAT2: rats treated with integrated combinations of T and A extracts integrated extract combinations.

Feed intake increased in all OVX rats compared to the control group (Figure 3A; *p* < 0.05), but no significant differences were found among the treatment groups (Figure 3A; *p* > 0.05). Similarly, OVX rats showed increased weight gain compared to the control group (Figure 3B; *p* < 0.05), though no significant differences were observed among the treatment groups (Figure 3B; *p* > 0.05).

### 3.3. Serum Biochemical Parameters

**Figure 4:**
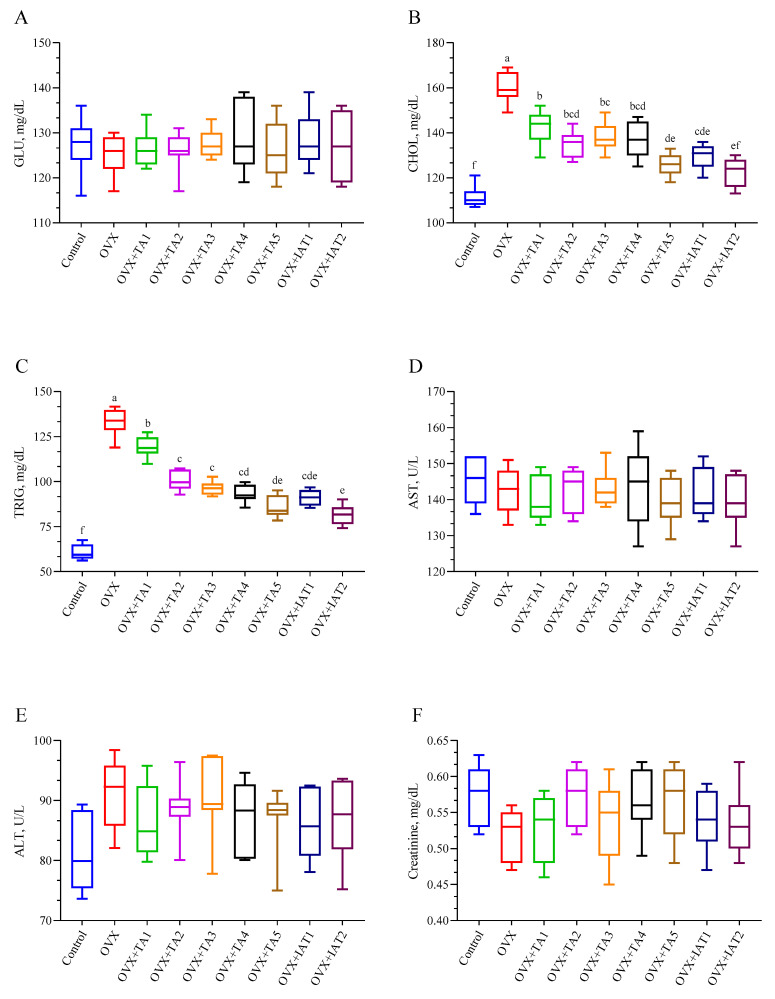
Effects of *Trigonella foenum-graecum* (TFG) and *Asparagus racemosus* (AR) combinations on glucose (**A**), cholesterol (**B**), triglyceride (**C**), aspartate aminotransferase (**D**), alanine transaminase (**E**), and creatinine (**F**) levels in OVX rats (n = 7). Whisker plots show median and min–max values; different letters (a–f) denote statistical differences (ANOVA, Tukey’s test). Group abbreviations: Control: on-ovariectomized rats without any treatment; OVX (Ovariec-tomy): Rats that underwent ovariectomy; OVX+TA1-TA5: rats treated with different dose combinations of Trigonella foenum-graecum (T) and Asparagus racemosus (A), OVX+IAT1-IAT2: rats treated with integrated combinations of T and A extracts integrated extract combinations.

No significant differences were found in GLU, AST, ALT, and creatinine levels among the control, OVX, and treatment groups (Figure 4A,D–F; *p* > 0.05). However, CHOL and TRIG levels were significantly elevated in the OVX group and were effectively reduced with treatment, with the most pronounced decrease observed in the IAT1 and IAT2 groups (Figure 4; *p* < 0.0001).

**Figure 5:**
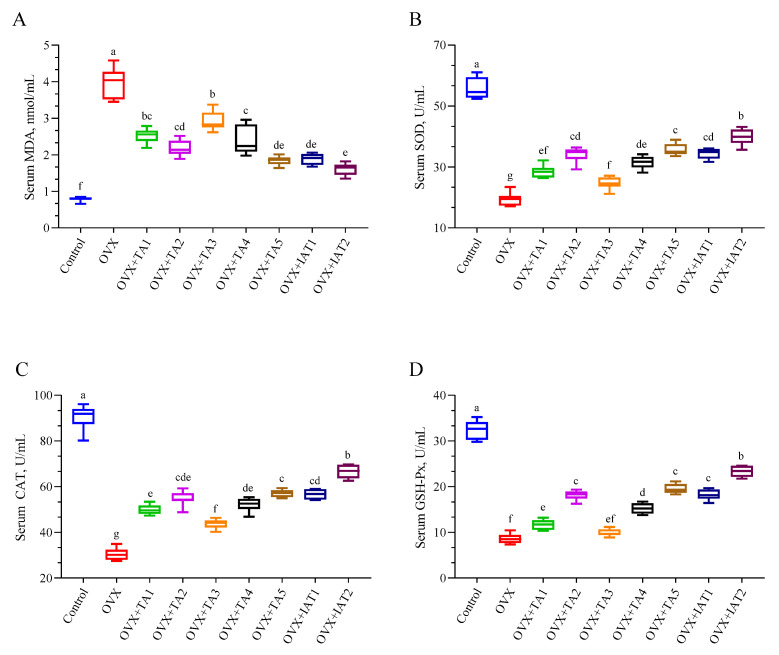
Effects of *Trigonella foenum-graecum* (TFG) and *Asparagus racemosus* (AR) combinations on MDA (**A**), SOD (**B**), CAT (**C**), and GSH-Px (**D**) in OVX rats (n = 7). Whisker plots show median and min–max values; different letters (a–g) indicate statistical differences (ANOVA, Tukey’s test). Group abbreviations: Control: on-ovariectomized rats without any treatment; OVX (Ovariectomy): Rats that under-went ovariectomy; OVX+TA1-TA5: rats treated with different dose combinations of Trigonella foenum-graecum (T) and Asparagus racemosus (A), OVX+IAT1-IAT2: rats treated with integrated combinations of T and A extracts integrated extract combinations.

The OVX group exhibited significantly higher serum MDA levels compared to the control group (Figure 5A; *p* < 0.0001) and reduced antioxidant enzyme activities (SOD, CAT, and GSH-Px) (Figure 5B–D; *p* < 0.0001). Treatment with test products significantly lowered MDA levels (*p* < 0.0001), with the greatest reduction observed in the IAT2 group (*p* < 0.0001). Similarly, the IAT2 group showed the most substantial restoration of serum SOD (*p* < 0.0001), CAT (*p* < 0.0001), and GSH-Px activities compared to the OVX group (*p* < 0.0001).

**Figure 6:**
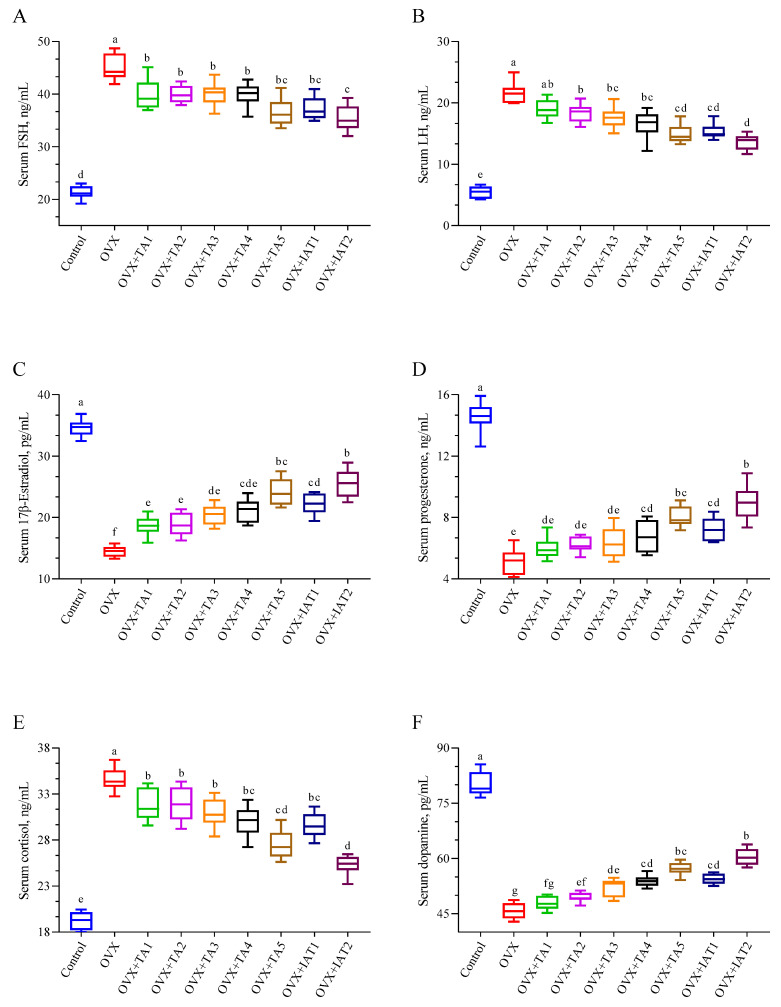
Effects of *Trigonella foenum-graecum* (TFG) and *Asparagus racemosus* (AR) combinations on FSH (**A**), LH (**B**), 17β-estradiol (**C**), progesterone (**D**), cortisol (**E**), and dopamine (**F**) in OVX rats (n = 7). Whisker plots show median and min–max values; different letters (a–g) indicate statistical differences (ANOVA, Tukey’s test). Group abbreviations: Control: on-ovariectomized rats without any treatment; OVX (Ovariectomy): Rats that un-der-went ovariectomy; OVX+TA1-TA5: rats treated with different dose combinations of Trigonella foenum-graecum (T) and Asparagus racemosus (A), OVX+IAT1-IAT2: rats treated with integrated combinations of T and A extracts integrated extract combinations.

Serum levels of FSH, LH, and cortisol were significantly higher in the OVX group (*p* < 0.0001; Figure 6A,B,E) but were notably reduced in treatment groups, with the greatest decrease observed in the IAT2 group (*p* < 0.0001; Figure 6A,B,E). Conversely, 17β-Estradiol, progesterone, and dopamine levels, which were significantly lower in the OVX group compared to controls (*p* < 0.0001; Figure 6C,D,F), were restored following with treatment, with the most pronounced effect in the IAT2 group, followed by IAT1.

### 3.4. Kisspeptin, NKB, TRPV1, c-FOS, BDNF, and GnRH Levels

**Figure 7:**
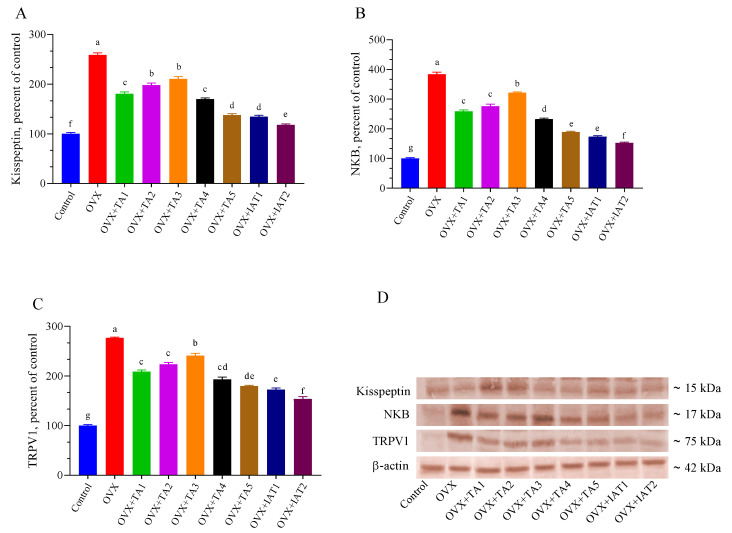
Effects of *Trigonella foenum-graecum* (TFG) and *Asparagus racemosus* (AR) combinations on Kisspeptin (**A**), NKB (**B**), TRPV1 (**C**) protein levels and representative Western blot bands (**D** and Figure S1) in OVX rats (n = 3). Densitometric analysis of Western blot bands normalized to β-actin ensured equal protein loading. Error bars represent the standard error of the mean; different letters (a–g) indicate statistical differences (ANOVA, Tukey’s test). Group abbreviations: Control: on-ovariectomized rats without any treatment; OVX (Ovariec-tomy): Rats that un-der-went ovariectomy; OVX+TA1-TA5: rats treated with different dose combinations of Trigonella foenum-graecum (T) and Asparagus racemosus (A), OVX+IAT1-IAT2: rats treated with integrated combinations of T and A extracts integrated extract combinations.

**Figure 8:**
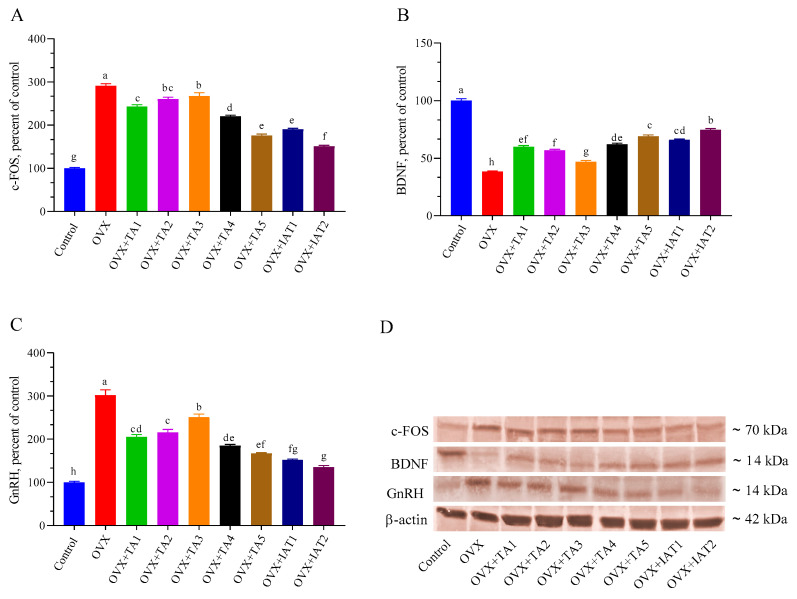
Effects of *Trigonella foenum-graecum* (TFG) and *Asparagus racemosus* (AR) combinations on c-FOS (**A**), BDNF (**B**), GnRH (**C**) protein levels and representative Western blot bands (**D** and Figure S2) in OVX rats (n = 3). Densitometric analysis of Western blot bands normalized to β-actin ensured equal protein loading. Error bars indicate the standard error of the mean; different letters (a–h) represent statistical differences (ANOVA, Tukey’s test). Group abbreviations: Control: on-ovariectomized rats without any treatment; OVX (Ovariectomy): Rats that un-der-went ovariectomy; OVX+TA1-TA5: rats treated with different dose combinations of Trigonella foenum-graecum (T) and Asparagus racemosus (A), OVX+IAT1-IAT2: rats treated with integrated combinations of T and A extracts integrated extract combinations.

Brain levels of Kisspeptin, NKB, and TRPV1 were significantly higher in the OVX group compared to the control group (Figure 7; *p* < 0.0001) but were effectively restored by IAT2 treatment (Figure 7B,C; *p* < 0.0001 for all). Similarly, c-FOS and GnRH levels were significantly elevated, while BDNF levels were significantly reduced in OVX rats compared to controls (Figure 8; *p* < 0.0001 for all). These alterations were reversed in the treated groups, with the most pronounced effect observed in the IAT2 group.

## 4. Discussion

Menopause is often accompanied by distressing symptoms such as hot flashes, stress, anxiety, and poor sleep, significantly impacting quality of life. Additional risks include heart disease and bone loss [3,6]. These symptoms are primarily linked to the decline in estrogen and progesterone production by the ovaries [26]. Although HRT is the most effective and reliable treatment option for the management of menopausal symptoms, long-term HRT carries an increased risk of hormone-related cancers and cardiovascular disease. This study highlights the protective effects of a novel integrated extract of AR and TFG seeds (IAT), a phytoestrogen, in managing menopausal symptoms. Using an OVX rat model of menopause, we demonstrated that IAT effectively mitigates menopausal symptoms and modulates associated molecular pathways. We observed that OVX rats showed reduced serum E2 and progesterone levels, which were significantly restored with IAT, particularly in the higher-dose IAT2 group. Serum FSH, LH, and OVX-induced increases in body and tail temperatures, indicative of hot flashes, were significantly reduced by IAT. This was associated with decreased levels of neuronal peptides such as Kisspeptin, NKB, TRPV1, c-FOS, and GnRH, which mediate hot flashes. Elevated serum cholesterol and triglyceride levels in OVX rats were restored with IAT. Additionally, IAT reduced oxidative stress, enhanced antioxidant enzyme activity, and restored neuroprotective peptides such as BDNF and dopamine, which regulate mood, emotions, and sleep. Notably, the integrated extract (IAT) was more effective than individual Asparagus and Trigonella extracts, with the IAT2 group showing the strongest effects. Importantly, all plant extracts used in this study were found to be safe, with no adverse effects or abnormalities in serum biochemical parameters.

The induction of experimental menopause by bilateral ovariectomy in reproductively competent young rats is a well-established and widely utilized model in menopause-related discovery and translational research. This procedure leads to a significant reduction in serum estradiol levels, typically observed within 1–2 weeks [42,43]. Both TFG and AR have been extensively employed in traditional medicine to address women’s health issues and are recognized for their estrogenic properties. Previous studies have demonstrated that AR extracts effectively alleviate vasomotor symptoms of menopause in both clinical settings and experimental rat models [1,35,44,45]. Similarly, TFG seed extracts have been shown to reduce hot flash symptoms, improve sleep, and alleviate depression in menopausal women and experimental menopause models [35,46,47]. In the present study, we observed elevated FSH and LH levels in OVX rats, which were significantly reduced following the administration of TFG, AR, and IAT extracts. Among these, the most pronounced effects were seen in the IAT2 group, suggesting a potential synergistic interaction between AR and TFG in restoring hormonal equilibrium. Our findings align with previous reports indicating that AR modulates the secretion of reproductive hormones within the HPG axis [45]. The beneficial effects of these phytochemicals on menopausal symptoms appear to be driven by multiple interconnected mechanisms. A primary factor is its estrogenic activity, largely attributed to the bioactive components of AR and TFG. This activity plays a crucial role in restoring hormonal balance, which, in turn, regulates key physiological processes such as thermoregulation, lipid metabolism, and oxidative stress [28,45,46]. TFG contains phytoestrogens, such as diosgenin, which interact with estrogen receptors and help alleviate menopausal symptoms [48,49,50]. These compounds mimic endogenous estrogens, thereby reducing the hormonal disruptions commonly observed after menopause. The estrogenic effects of AR have been demonstrated in various animal models, where oral administration of its root extract influenced mammary gland and genital organ development, particularly during pregnancy [51]. AR has also been used to correct hormonal imbalances and mitigate menopausal symptoms, with its primary bioactive constituents, steroidal saponins, particularly shatavarins I–IV, believed to contribute to these effects [52]. Studies have shown that AR treatment increases serum levels of 17β-estradiol, free testosterone, and progesterone while concurrently reducing FSH and steroid hormone-binding globulin [47].

One of the hallmark symptoms of menopause is hot flashes, which are linked to dysregulated thermoregulation due to estrogen deficiency. Estrogen plays a crucial role in modulating hypothalamic Kisspeptin, Neurokinin B (NKB), and Dynorphin (KNDγ) neurons, which regulate GnRH and LH release. The estrogen deficiency in OVX rats results in heightened Kisspeptin and NKB activity, leading to excessive GnRH and LH secretion, which in turn disrupts thermoregulation and triggers hot flashes [12,53,54]. Kisspeptin, a potent stimulator of GnRH release, is known to be highly responsive to sex steroid feedback in both animal models and humans [53]. Similarly, neurokinin B (NKB) plays a key stimulatory role in the secretion of GnRH and LH [54]. Somatic hypertrophy of KNDγ neurons, along with increased expression of NKB and kisspeptin genes, has been observed in postmenopausal women [12] and ovariectomized (OVX) rats [8,55]. In the current study, we detected elevated protein expression levels of kisspeptin, NKB, and GnRH in the brain tissues of OVX rats. This increase was mitigated following the administration of plant extracts, with the highest efficacy observed in the IAT group at the higher concentration. Additionally, we noted elevated protein expression levels of TRPV1 and c-FOS in OVX rats, consistent with previous findings [21]. These levels were restored to near-normal values upon treatment with the plant extracts. Hot flashes are characterized by an increase in body temperature, often accompanied by sweating and peripheral vasodilation. Similar thermoregulatory changes have been documented in rodent models, such as OVX rats, where elevated skin and body temperatures were reported approximately two weeks post-ovariectomy, mirroring the thermoregulatory disruptions observed in humans [56].

Another potential explanation is the antioxidant properties of AR and TFG, which likely contribute to reduced oxidative stress and inflammation in OVX rats. The increase in oxidative stress markers following ovariectomy has been well documented [57,58,59,60], and the ability of IAT to restore antioxidant enzyme activity suggests a protective mechanism against oxidative damage. Additionally, the observed reductions in cholesterol and triglyceride levels in the IAT-treated groups could be due to the lipid-modulating effects of TFG, which have been previously shown to regulate lipid metabolism and improve insulin sensitivity [61,62]. Estrogen deficiency is associated with increased oxidative stress, leading to neuronal damage and impaired cognitive function. This oxidative imbalance is characterized by elevated levels of MDA and nitric oxide (NO), along with reduced activities of key antioxidant enzymes (SOD, GSH-Px, and CAT) [63]. In our study, IAT treatment significantly reduced MDA levels while enhancing SOD, GSH-Px, and CAT activities, indicating strong antioxidant properties. These effects can be attributed to the bioactive phytochemicals in AR and TFG, including flavonoids and saponins, which are known to scavenge reactive oxygen species (ROS) and upregulate endogenous antioxidant defenses [49,61]. Furthermore, AR has been shown to exert neuroprotective effects by enhancing brain-derived neurotrophic factor (BDNF) expression, which is crucial for neuronal survival, synaptic plasticity, and cognitive function. The observed increase in BDNF levels in IAT-treated OVX rats further supports its role in neuroprotection.

The cessation of estrogen production during menopause is commonly associated with increased body weight and obesity-related disorders, including cardiovascular disease [64,65]. In the present study, serum triglyceride and cholesterol levels were elevated in OVX rats but were significantly reduced in animals treated with TFG, AR, and IAT extracts. Similar findings have been reported in previous studies, where ovariectomy in rats led to increased plasma levels of total cholesterol, HDL-cholesterol, LDL-cholesterol, and triglycerides. These levels were subsequently reduced following treatment with “Njansang” oil, known for its antioxidant and anti-inflammatory properties [66]. TFG has been shown to effectively regulate lipid profiles in experimental models, reducing serum triglycerides, total cholesterol, and hepatic lipid concentrations [61,62]. These findings highlight its lipid-modulating potential, which is further supported by the results of our study. The marked reduction in estrogen levels during menopause has been associated with increased oxidative stress, disrupting the balance between antioxidants and oxidants. Similar disruptions have been observed in mammals following ovariectomy or ovariohysterectomy [57].

The reduction in progesterone and estrogen levels during surgical or natural menopause has been shown to disrupt neurochemical communication in the brain [59,67,68], resulting in decreased levels of neurotransmitters such as dopamine and serotonin [69]. Consistent with these findings, this study observed reduced dopamine levels in OVX rats, likely linked to lower serum 17β-estradiol and progesterone levels. This deficit was effectively restored with IAT treatment. Saponins from AR root extract have been reported to exhibit antidepressant [70], nootropic, and anti-amnesic properties in experimental models [71], likely mediated through the inhibition of acetylcholine and monoamine metabolizing enzymes under in vitro conditions [72]. Additionally, AR demonstrates anxiolytic effects through modulation of the GABAergic system or amygdala 5-HT2A-mediated serotonergic pathways, making it a promising candidate for anxiety management [73]. Similarly, flavonoids derived from TFG seeds, such as serotonin, are known to enhance monoamine neurotransmitter levels in the brain [74]. These compounds have also been shown to ameliorate cognitive deficits in streptozotocin-induced diabetic rats [75]. The combined properties of TFG and AR may contribute to the restoration of neurochemical balance and improved neurological function observed in our study.

Decreased levels of BDNF observed after menopause were restored by hormone therapy indicative of the modulatory effect of estradiol or progesterone on BDNF expression [76]. BDNF expression is reduced in rats by ovariectomy with decreased hippocampal functions [59,77,78]. We observed a significant reduction in BDNF expression in the brain tissue of OVX rats, which was significantly restored by IAT extract, which is in agreement with a previous study where fenugreek seed extract supplementation significantly increased BDNF expression in the brain of OVX rats [79].

The selection of TFG and AR doses in this study was based on previous human and animal studies [1,37]. A study indicates that fenugreek extract, when administered at doses ranging from 500 to 1000 mg per day, significantly improves postmenopausal symptoms, hormonal balance, and vasomotor symptoms [37]. Additionally, a clinical study using formulations containing AR at doses of approximately 100 mg per day has demonstrated significant benefits in reducing menopausal symptoms [1]. Given these findings, we tested a range of doses in the OVX rat model to determine the optimal dose-response effects. However, while our study demonstrates the effectiveness of different doses of IAT, we primarily focused on the supplemental effects relative to the OVX control group rather than conducting a detailed dose-response analysis of individual TFG and AR components. Future studies should further explore the dose-dependent effects of TFG and AR individually to better understand their specific contributions to the observed outcomes. Additionally, investigating whether a lower or higher range of doses could yield comparable or superior effects would provide further insights into the therapeutic potential of these phytoestrogens.

While this study provides strong evidence supporting the beneficial effects of IAT in alleviating menopausal symptoms in ovariectomized rats, there are several limitations to consider. First, this study was conducted using an animal model, which may not fully replicate the complexity of human menopause. Although ovariectomized rats serve as a widely accepted model for menopause research, differences in metabolism, hormonal regulation, and physiological responses between humans and rodents must be considered when extrapolating the findings. Another limitation is the duration of this study. The intervention period was limited to eight days, which may not fully capture the long-term effects or safety profile of the extracts. Future studies should explore the prolonged administration of IAT to assess its efficacy and potential side effects over an extended period.

## 5. Conclusions

This study underscores the significant advantages of combining *Trigonella foenum-graecum* and *Asparagus racemosus* extracts over using each individually in alleviating menopausal symptoms in ovariectomized rats. The combination demonstrated efficacy in restoring reproductive hormone levels, regulating lipid profiles, enhancing neuronal signals such as dopamine and BDNF, reducing oxidative stress, and bolstering antioxidant defense systems. Future clinical trials in humans will be essential to validate these findings and establish the IAT preparation as a safer, more effective alternative to hormone replacement therapy for managing menopausal symptoms, including hot flashes, with fewer adverse effects.

## Figures and Tables

**Table 1:** Composition of the standard diet.

Ingredients	%
Casein	20.00
Corn starch	57.95
Sucrose	5.00
Vegetable oil	7.00
Beef tallow	–
Cellulose	5.00
Mineral premix ^a^	3.50
Vitamin premix ^b^	1.00
L-cysteine	0.30
Choline bitartrate	0.25

^a^ Modified AIN-93G-MX. ^b^ AIN-93-VX (No: 310025).

## Data Availability

The data presented in this study are available on request from the corresponding author.

## Supplementary Materials

The following supporting information can be downloaded at: https://www.mdpi.com/article/10.3390/antiox14030355/s1, Figure S1. Full immunoblots related to Figure 6 in the main text: The effects of Trigonella foenum-graecum (TFG) and Asparagus racemosus (AR) combinations on brain tissue Kisspeptin (A1, A2, A3), NKB (B1, B2, B3), and TRPV1 (C1, C2, C3) are shown. The densitometric analysis of the relative intensity of Western blot bands, normalized to β-actin for ensuring equal protein loading, was performed relative to the normal control group (D1, D2, D3). Black dotted rectangles highlight the results presented in Figure 6 of the main text. Molecular weight (M.W.) markers are indicated in kilodaltons (kDa). Group abbreviations: Control, Ovariectomy (OVX), OVX+TA1-TA5 (different TFG+AR combinations), OVX+IAT1–IAT2 (integrated TFG+AR extracts). Figure S2. Full immunoblots related to Figure 7 in the main text: The effects of Trigonella foenum-graecum (TFG) and Asparagus racemosus (AR) combinations on brain tissue c-FOS (A1, A2, A3), BDNF (B1, B2, B3), and GnRH (C1, C2, C3) are shown. The densitometric analysis of the relative intensity of Western blot bands, normalized to β-actin for ensuring equal protein loading, was performed relative to the normal control group (D1, D2, D3). Black dotted rectangles highlight the results presented in Figure 7 of the main text. Molecular weight (M.W.) markers are indicated in kilodaltons (kDa). Group abbreviations: Control, Ovariectomy (OVX), OVX+TA1-TA5 (different TFG+AR combinations), OVX+IAT1–IAT2 (integrated TFG+AR extracts).
