# Supplementary material for: Effects of Integrated Extracts of Trigonella foenum-graecum and Asparagus racemosus on Hot Flash-like Symptoms in Ovariectomized Rats
Source: Antioxidants (Basel). 2025 Mar 18;14(3):355. doi: 10.3390/antiox14030355 (PMC11939183; doi:10.3390/antiox14030355)
Supplement: Supplementary file 1 [file antioxidants-14-00355-s001.zip › antioxidants-3454168-supplementary.pdf]

Supplementary Materials

Effects of Integrated Extracts of *Trigonella Foenum-Graecum* and *Asparagus Racemosus* on Hot Flash-Like Symptoms in Ovariectomized Rats

Fusun Erten[1], Besir Er[2], Ramazan Ozmen[3], Muhammed Tokmak[3], Ebru Gokdere[4], Cemal Orhan[3], Abhijeet A. Morde[5], Muralidhara Padigaru [5], Kazim Sahin[3,*]

[1] Department of Veterinary Science, Pertek Sakine Genc Vocational School, Munzur University, 62500, Tunceli, Turkiye; fusunerten@munzur.edu.tr (F.E.)

[2] Department of Biology, Faculty of Science, Firat University, 23119, Elazig, Turkiye; ber@firat.edu.tr (B.E.)

[3] Department of Animal Nutrition, Faculty of Veterinary Medicine, Firat University, 23119, Elazig, Turkiye; : ksahin@firat.edu.tr (K.S.); corhan@firat.edu.tr (C.O.); rozmen@firat.edu.tr (R.O.); 211304205@firat.edu.tr (M.T.)

[4] Department of Physiology, Faculty of Medicine, Firat University, 23119, Elazig, Turkiye; egokdere@firat.edu.tr (E.G.)

[5] Research and Development, OmniActive Health Technologies Co., Ltd., Mumbai 400013, India; a.morde@omniactives.com (A.A.M.); m.padigaru@omniactives.com (M.P.)

* Correspondence: ksahin@firat.edu.tr; Tel.: +90-532-747-3506 or +90-424-237-0000 (ext. 3938)

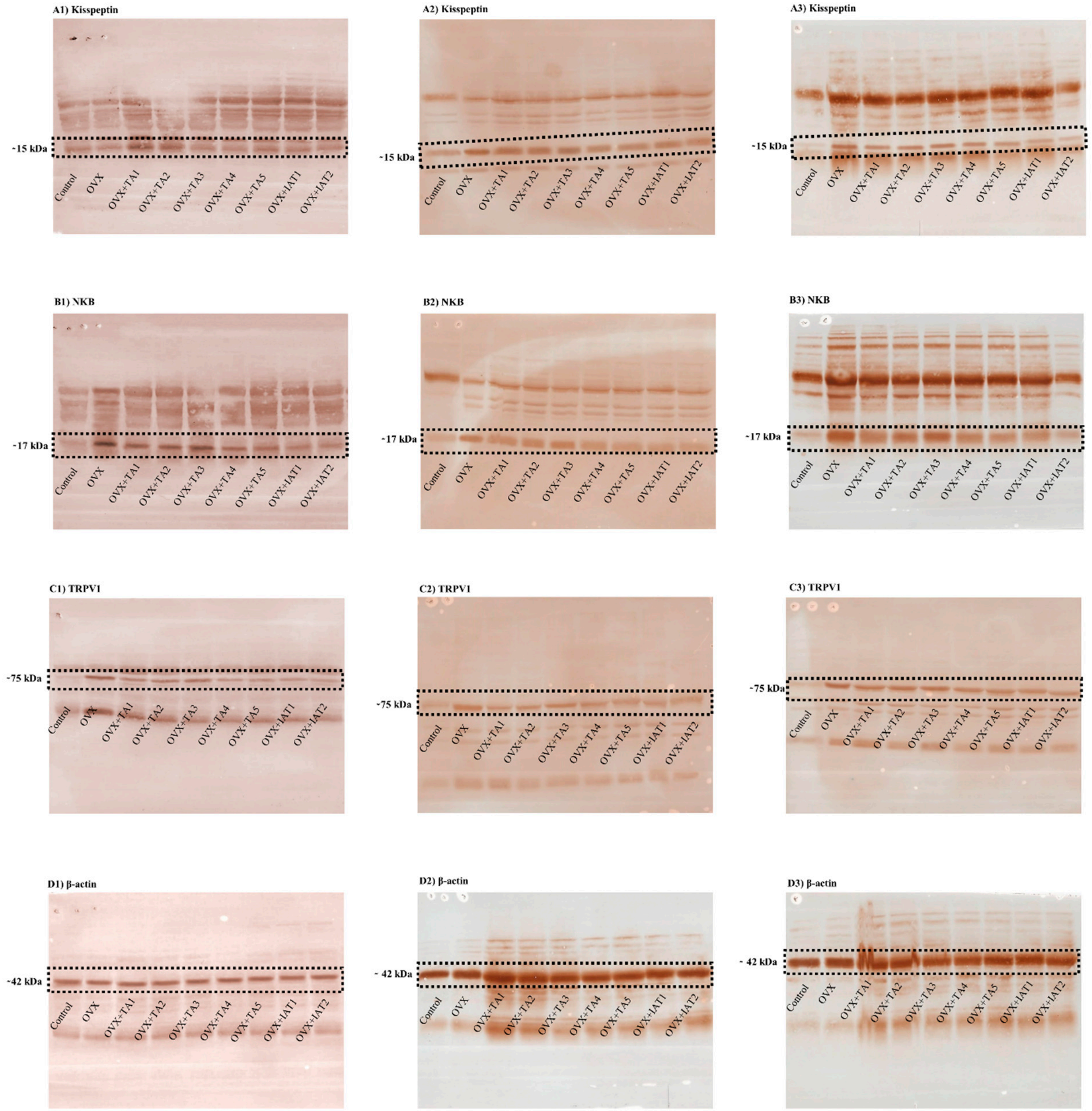

Figure S1. Full immunoblots related to Figure 6 in the main text: The effects of Trigonella foenum-graecum (TFG) and Asparagus racemosus (AR) combinations on brain tissue Kisspeptin (A1, A2, A3), NKB (B1, B2, B3), and TRPV1 (C1, C2, C3) are shown. The densitometric analysis of the relative intensity of Western blot bands, normalized to β-actin for ensuring equal protein loading, was performed relative to the normal control group (D1, D2, D3). Black dotted rectangles highlight the results presented in Figure 6 of the main text. Molecular weight (M.W.) markers are indicated in kilodaltons (kDa). Group abbreviations: Control, Ovariectomy (OVX), OVX+TA1–TA5 (different TFG+AR combinations), OVX+IAT1–IAT2 (integrated TFG+AR extracts).

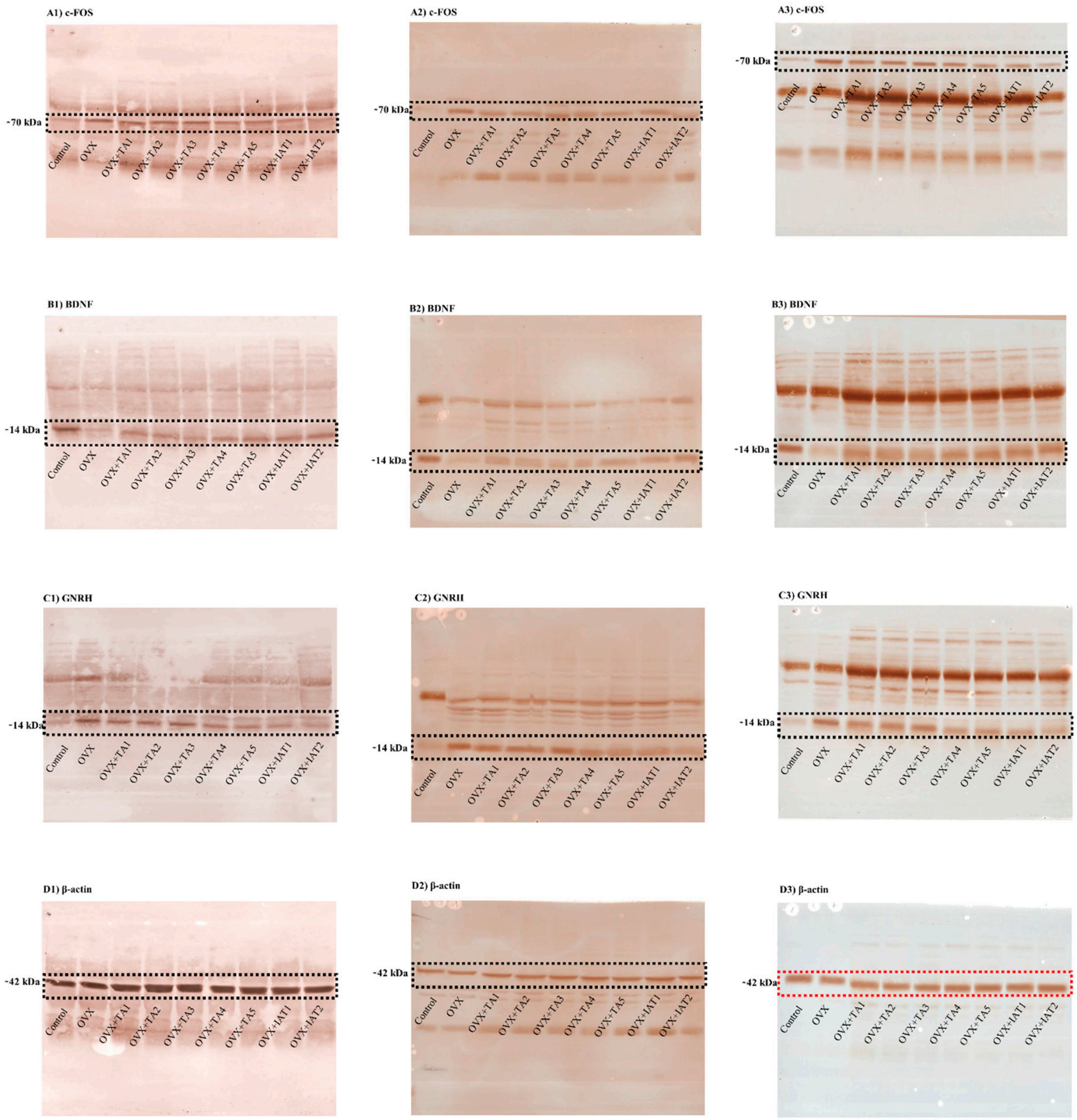

Figure S2. Full immunoblots related to Figure 7 in the main text: The effects of Trigonella foenum-graecum (TFG) and Asparagus racemosus (AR) combinations on brain tissue c-FOS (A1, A2, A3), BDNF (B1, B2, B3), and GnRH (C1, C2, C3) are shown. The densitometric analysis of the relative intensity of Western blot bands, normalized to β-actin for ensuring equal protein loading, was performed relative to the normal control group (D1, D2, D3). Black dotted rectangles highlight the results presented in Figure 7 of the main text. Molecular weight (M.W.) markers are indicated in kilodaltons (kDa). Group abbreviations: Control, Ovariectomy (OVX), OVX+TA1–TA5 (different TFG+AR combinations), OVX+IAT1–IAT2 (integrated TFG+AR extracts).
